# The Role of Abnormal Placentation in Congenital Heart Disease; Cause, Correlate, or Consequence?

**DOI:** 10.3389/fphys.2018.01045

**Published:** 2018-08-07

**Authors:** Jennifer A. Courtney, James F. Cnota, Helen N. Jones

**Affiliations:** ^1^Molecular and Developmental Biology Graduate Program, Cincinnati Children's Hospital Medical Center, Cincinnati, OH, United States; ^2^Division of General Pediatric and Thoracic Surgery, Center for Fetal and Placental Research, Cincinnati Children's Hospital Medical Center, Cincinnati, OH, United States; ^3^Heart Institute, Cincinnati Children's Hospital Medical Center, Cincinnati, OH, United States

**Keywords:** placentation, congential heart defects, hemodynamics, heart development, molecular mechanisms

## Abstract

Congenital heart disease (CHD) is the most common birth defect, affecting ~1% of all live births (van der Linde et al., 2011). Despite improvements in clinical care, it is the leading cause of infant mortality related to birth defects (Yang et al., 2006) and burdens survivors with significant morbidity (Gilboa et al., 2016). Furthermore, CHD accounts for the largest proportion (26.7%) of birth defect-associated hospitalization costs—up to $6.1 billion in 2013 (Arth et al., 2017). Yet after decades of research with a primary focus on genetic etiology, the underlying cause of these defects remains unknown in the majority of cases (Zaidi and Brueckner, 2017). Unexplained CHD may be secondary to undiscovered roles of noncoding genetic, epigenetic, and environmental factors, among others (Russell et al., 2018). Population studies have recently demonstrated that pregnancies complicated by CHD also carry a higher risk of developing pathologies associated with an abnormal placenta including growth disturbances (Puri et al., 2017), preeclampsia (Auger et al., 2015; Brodwall et al., 2016), preterm birth (Laas et al., 2012), and stillbirth (Jorgensen et al., 2014). Both the heart and placenta are vascular organs and develop concurrently; therefore, shared pathways almost certainly direct the development of both. The involvement of placental abnormalities in congenital heart disease, whether causal, commensurate or reactive, is under investigated and given the common developmental window and shared developmental pathways of the heart and placenta and concurrent vasculature development, we propose that further investigation combining clinical data, *in vitro, in vivo*, and computer modeling is fundamental to our understanding and the potential to develop therapeutics.

## Concurrent development of the heart and placenta

The heart and placenta develop concurrently, with heart tube specification occurring at days 16–21 and a rudimentary villous tree forming by day 21 of gestation (Schleich, 2002; Kaufmann et al., 2004; Khong, 2004; Linask, 2013; Tyser et al., 2016).

**Figure 1:**
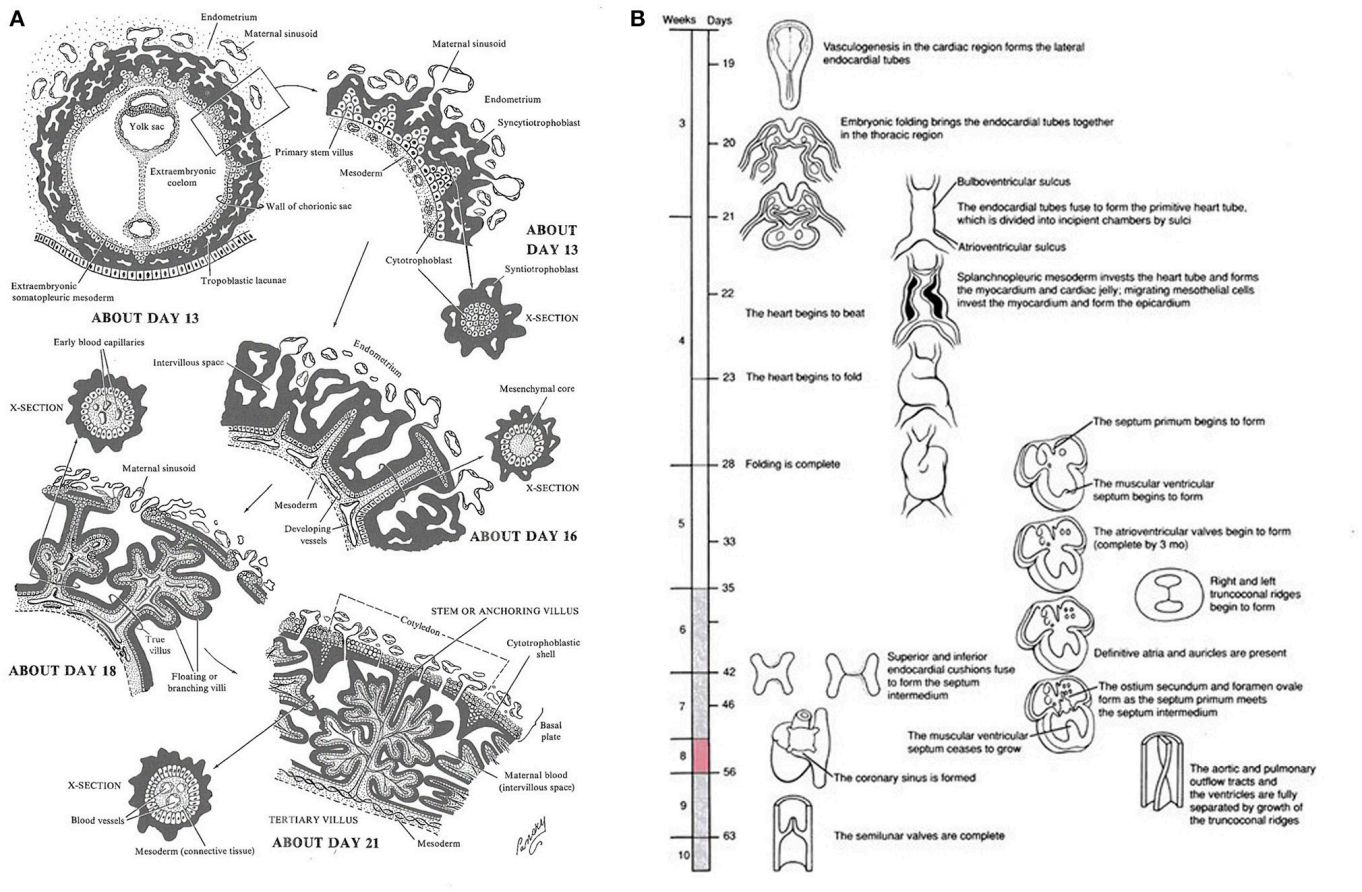
**(A)** Timeline of human early placental development: Adapted from Lifemap Discovery (Edgar et al., 2013), “At the beginning of week 3 primary stem villi, consisting of a cytotrophoblast core covered by a syncytial layer, appear. Extraembryonic mesodermal cells or cytotrophoblast penetrate the core of the primary villi and grow in the direction of the decidua to form secondary stem villi and by the end of week 3 the mesodermal cells differentiate into blood cells and small blood vessels, forming the villous capillary system, and creating tertiary villi. By week 4, capillaries in the tertiary villi contact capillaries developing in the mesoderm of the chorionic plate and in the connecting stalk, eventually contact the intraembryonic circulatory system, and connect the placenta and the embryo. Thus, in week 4, when the heart begins to beat, the placental villous system is able to supply the embryo with oxygen and nutrients.” **(B)** Timeline of human embryonic heart development: Adapted from Lifemap Discovery (Edgar et al., 2013). “The human heart develops on day 18 or 19 following fertilization. In response to induction signals from the underlying endoderm, the mesoderm in the cardiogenic area forms the cardiogenic cords. A hollow center forms within the cords, giving rise to the endocardial tubes. With lateral folding of the embryo, the paired endocardial tubes approach each other and fuse into a single tube called the primitive heart tube. The primitive heart tube develops into five distinct unpaired regions and begins to pump blood”.

Approximately 12 days post conception, the placenta comprises a layer of extra-embryonic mesoderm and two trophoblast populations, a core of cytotrophoblast cells covered by a layer of syncytiotrophoblast. Over the next 3–8 days the mesenchymal cell cores of the primary villi transform into first hemangiogenic precursor cells (Huppertz and Peeters, 2005). The villous trophoblast at this early stage of development is paramount in regulating the development of the placental vasculature with several components of key signaling pathways only expressed in the trophoblast, Figure 1A (Rossant and Cross, 2001). At the same time the heart forms, first as bilateral cardiogenic plates of mesoderm, then as a primitive heart tube as the two plates fuse during gastrulation (Gittenberger-de Groot et al., 2005; Lindsey et al., 2014). By day 20, the heart tube beats steadily, and includes a single ventricle and outflow tract (Schleich, 2002) Figure 1B (Gilbert and Barresi, 2016).

**Figure 2:**
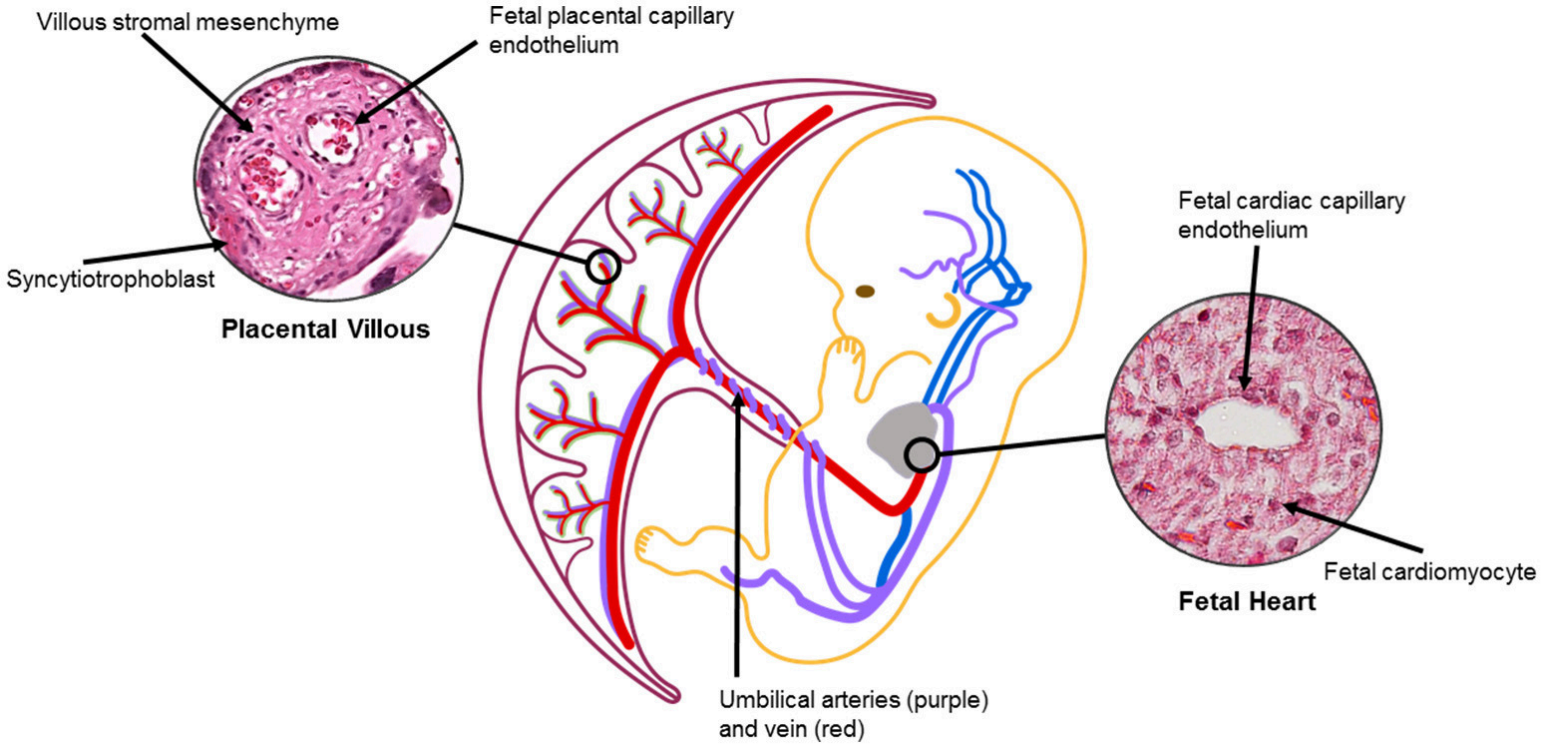
The placental and heart are connected via fetal vasculature. Signaling between trophoblast/endothelial and/or cardiomyocyte/endothelial cells may impact fetal vasculature, leading to changes in placental and heart structures (Jia et al., 2018). Oxygenation, flow, cell crosstalk can also affect organ development and remodeling via vascular changes throughout gestation. Placental villous image from Boyd Collection Centre for Trophoblast Research at the University of Cambridge. https://www.trophoblast.cam.ac.uk/Resources/ndp-index^1^.

Vascularization of the human placenta occurs by local *de-novo* vasculogenesis within the mesenchymal core of the secondary villi. This occurs prior to infiltration of fetal vessels or blood into the placenta with the progenitor cells derived directly from placental mesenchymal cells (Demir et al., 1989). Shortly after this, Hofbauer cells (macrophages of placental origin) develop inside the villous core in the vicinity of the vasculogenic precursor cells suggesting a putative paracrine role for these cells during the early stages of placental vasculogenesis (Demir and Erbengi, 1984; Cervar et al., 1999). During very early placental development, Vascular endothelial growth factor (VEGF) is highly expressed in cytotrophoblast cells and also in Hofbauer cells. On the other hand, the respective receptors, Flt-1 and Flk-1 are expressed on the vasculogenic and angiogenic precursor cells (Charnock-Jones et al., 2004). With this constellation an increase in the expression of VEGF and its receptors may orchestrate the temporal and spacial regulation of the differentiation and maturation of villous vascularization (Castellucci et al., 2000; Kingdom et al., 2000). Concurrently, heart looping occurs during week 4 and is controlled by gene regulatory networks for left-right patterning and local gradients (Lenhart et al., 2013; Sylva et al., 2014). Septa and valves form via tissue folding and cardiac cushions that undergo epithelial-mesenchyme transition during week 5 (Anderson et al., 2003). Cardiac vessel morphogenesis occurs concurrently via inductive signaling of epicardium from underlying myocardium (Lin et al., 2012). Fetal vascular development and circulation is established between 25 and 60 days and despite an extensive capillary network within early villi, there is little evidence of fetal-placental circulation until late first trimester (Thornburg and Louey, 2013). Once established, signaling between the placenta and fetal organs via the fetal-placental circulation may impact growth and remodeling of both the fetal heart and the placenta, which, in the placenta occurs throughout gestation until term (Figure 2).

## Common molecular pathways may direct both heart and placental development

Mechanisms of heart and placental development share common regulatory pathways, for example, cardiomyocyte specification and extra villous trophoblast invasion are both regulated by Notch and Wnt (De Falco et al, 2007; de la Pompa and Epstein, 2012). The endocardial cushion develops from neural crest cells to form heart valves and septa and requires similar genes as the placenta for proper formation and remodeling, including VegF and Connexin 43 (Maschhoff and Baldwin, 2000; Dor et al., 2001; Dunk et al., 2012).

Studies in both human and mouse demonstrate the effects of alterations in these genes and their associations with failure to develop appropriately. Connexin 43 mediates cell-cell interactions in cardiomyocytes, and aids in trophoblast fusion and intercellular placental communication (Maschhoff and Baldwin, 2000; Dunk et al., 2012). Babies with fetal growth restriction have lower levels of VCAM1 than those with normal growth trajectories (Rajashekhar et al., 2003). Targeted disruption of VCAM1 in mice leads to impaired placentation and fetal death, and severe abnormalities in the developing heart (Gurtner et al., 1995; Kwee et al., 1995), and deletion of the upstream gene FOXO1 attenuates VCAM1 expression and leads to similar outcomes (Ferdous et al, 2011). The loss of PPAR gamma in mice results in abnormal placental and cardiac development (Barak et al., 1999) with embryonic lethality mid-gestation. Using tetraploid embryos, PPAR gamma restoration to the inner cell mass did not overcome the placental or cardiac defects, however, importantly, when PPAR gamma was restored to the trophectoderm alone fetuses survive and cardiac malformations were absent.

Common molecular pathways also direct vasculogenesis and angiogenesis in both the heart and placenta. Loss of Cited2 in mice causes significant heart and fetal growth defects, disruption of left-right patterning, and fetal death (Bamforth et al., 2001, 2004; Lopes Floro et al., 2011). Placenta-specific mouse knockouts of Cited2 reveal disorganized labyrinthine placental vasculature by day E14.5 due to an impaired HIF-signaling response (Withington et al., 2006). Interestingly, targeted deletion of Cited2 in trophoblasts but not endothelial cells disrupted capillary patterning in the placenta (Moreau et al., 2014). Heart-specific knockout of Cited2 revealed similar myocardial and coronary vascular abnormalities (MacDonald et al., 2012), revealing that signaling by both trophoblasts and cardiomyocytes impacts local capillary growth and development. In contrast, a recent study by Rhee et al. (2018) identifies the necessity of endothelial cell signaling for regulation of cardiac development. The authors demonstrated left ventricular non-compaction when the chromatin remodeler Ino80 was knocked out in coronary vessel progenitor cells or in all endothelium, with a more severe heart phenotype in the endothelial-specific knockout. Unfortunately, this study did not include assessment of the placenta to identify if the endothelial-specific knockout also impacted placental development. Ino80 is a ubiquitous cell proliferation promoter, and disruption of endothelial cell proliferation pathways may underlie loss of conceptus or congenital defects with impacts on both heart and placenta.

Importantly, many murine studies published on genes necessary for heart development demonstrate a common phenotype, usually reported as embryonic lethal, that upon further analysis is due to malformation of a placental vascular tree, for example in the Hand1 and NKx2.5 null mice, driving the need to understand the roles of these genes in both placental and vascular development (Olson and Srivastava, 1996; Firulli et al., 2010). Recently, the Deciphering the Mechanisms of Developmental Disorders (DMDD) consortium demonstrated that 68% of the 103 mouse lines they investigated had placental phenotypes (Perez-Garcia et al., 2018). There was a strong correlation between heart and vascular abnormalities and placental defects; additionally, common genes affected heart and vascular morphology in the study.

**Figure 3:**
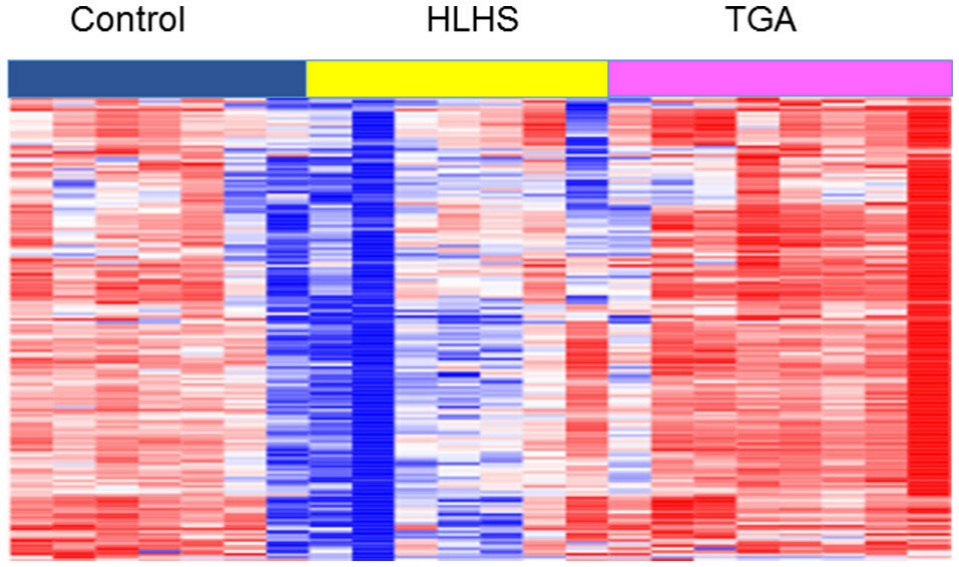
Heat map of RNA-sequencing profiles of term placentas from TGA, HLHS, and gestational-age matched controls from Cluster 1 of GoElite cluster analysis. Pathways significantly changed (*p* < 0.05) include determination of left/right symmetry, left/right patterning, heart looping, heart development, MHC protein complex, and ATP synthesis. A complete list of genes and pathways is included in Supplementary Material.

We have previously published impaired placental vascular tree development in one subtype of CHD in humans (Jones et al., 2015) and more recently demonstrated it in another (TGA). Furthermore, to investigate mechanisms of this disruption we performed RNA-sequencing on human placentas from fetuses with CHDs and compared them to gestational-age matched controls. Term placentas from TGA, HLHS, and control births were collected under institutional IRB approval. RNA was isolated from FFPE samples using RecoverAll™ Total Nucleic Acid Isolation Kit for FFPE. RNA-Sequencing using Ullumina HiSeq was performed by CCHMC and UC Core facilities. RNA-sequencing analysis was done using AltAnalyze version 2.0 (Kallisto for alignment) to identify differentially expressed genes and clustering performed using GoElite. Methods described in Supplementary Materials. Clustering analysis identified pathways involved in heart looping, left/right patterning and symmetry, muscle development, and ATP synthesis were significantly downregulated in the placentas of the Hypoplastic Left Heart Syndrome (HLHS) fetuses compared to controls (Figure 3 and Supplemental Table 1), indicating disruption to pathways associated with both heart and placental development.

## Placental/umbilical hemodynamics and heart development

Based on studies of blood flow and cardiac remodeling during development, it is possible that placental dysfunction may significantly contribute to the incidence of CHD. Whether the placenta contributes to the development of CHD or whether disruption of common developmental pathways contribute to placental dysfunction and CHDs is not known.

Developmental abnormalities which impair normal blood flow through the embryonic heart, including improper placentation and villous vascular tree development with resulting impairment of flow, may contribute to the occurrence of CHD in humans. Alteration in placental hemodynamics could impact both inflow via the umbilical vein and outflow via the umbilical artery. Midgett et al. (2017) disrupted blood flow to inflow and outflow tracts of chick embryos at HH18 (looped tubular beating heart), causing malformations of developing heart structures. Defects were shown to be flow dependent, with more restriction to the outflow tract causing more significant heart defects.

However, the study of feto-placental circulation by Doppler ultrasound in humans with CHD has resulted in conflicting results. Evidence of lower cerebral resistance, i.e., brain sparing, flow patterns have been demonstrated in multiple studies but likely varies by type of cardiac defect (Hahn et al., 2016; Ruiz et al., 2017). However, the majority of umbilical flow patterns fall within the normal range. As the antenatal diagnosis of CHD most commonly occurs in the second trimester of pregnancy, these clinically measurable tools are being applied long after the development of the cardiac defect and may not reflect early hemodynamic disturbances that could contribute to the etiology of CHD. Impaired blood flow may occur much earlier in pregnancy with extensive cardiac remodeling occurring to compensate for the disrupted flow.

Cardiac remodeling also occurs in response to hypoxia, which increases VegFR-3 (Flt4) expression and subsequently VegFC. This signaling pathway improves vasculogenesis, angiogenic sprouting, and fetal cardiac development (Helske et al., 2001). Gadd45G has been shown to be sensitive to hypoxia in the heart during ischemia, leading to apoptosis of cardiomyocytes, fibrosis, and left ventricular dysfunction (Lucas et al., 2015). Isoform Gadd45A in placenta is also induced by hypoxia and leads to poor migration of trophoblasts (Liu et al., 2014). Lower levels of both isoforms lead to better outcomes. Impaired placentation, fibrin deposition, and reduced placental vasculature impact the gas exchange capabilities of the placenta and therefore oxygen levels available to the fetus via the umbilical vein may be reduced which could also contribute to hypoxia-induced cardiac remodeling. Diminished umbilical venous oxygen levels on fetal MRI further support these hypotheses (Sun et al., 2015).

## Maternal and environmental effects on placental and heart development

During implantation, maternal signals direct embryonic cell invasion into the decidua, and maternal immunity determines depth of trophoblast invasion (Su and Fazleabas, 2015). Disruptions in uterine signaling or an overactive maternal immune response may limit invasion, leading to placental insufficiency and concurrent cardiac anomalies (Thornburg et al., 2010). Once the maternal-placental and fetal-placental circulations are established, the impact of the maternal or external environment, via the maternal circulation, may impact both placental and heart development directly (factors able to cross the placenta, e.g., flame retardant chemicals; Gorini et al., 2014) or may indirectly impact cardiac growth and development through abnormal signaling from the dysfunctional placenta. While existing understanding of CHD causation has largely focused on genetic etiologies (Zaidi and Brueckner, 2017), epidemiologic and experimental studies also support environmental contributions. Linask (2013) proposed that the maternal environment can also influence heart development. Multiple observational studies have linked multivitamins and folic acid supplementation to CHD (Botto et al., 2004). Proposed mechanisms are related to both DNA synthesis and methylation with numerous basic and translational studies addressing the question. Studies have associated maternal hyperlipidemia to an increased risk of CHD (Smedts et al., 2012; Wong et al., 2018), and maternal diabetes correlates strongly to certain CHDs in mouse studies (Hachisuga et al., 2015). Mouse studies indicate that folate deficiencies coupled with other environmental exposures (alcohol, lithium, Wnt3A deficiency) led to cardiac anomalies, while administering high folate levels in mice and humans before pregnancy prevented cardiac birth defects (Han et al., 2009; Serrano et al., 2010; Czeizel et al., 2015). Other maternal influences, such as hyperlipidemia and/or hyperglycemia, maternal obesity and maternal smoking have been associated with an increased risk of CHD (Alverson et al., 2011; Smedts et al., 2012).

## Fetal growth, CHD subtype

Current treatment for many complex CHDs is palliative surgery shortly after birth. Babies born small for gestational age (SGA) who also have a CHD exhibit higher mortality after the first surgical correction than those babies with normal birth weights (Best et al., 2017). We have identified significant abnormalities in placentas from fetuses with either Hypoplastic Left Heart Syndrome (HLHS) or Transposition of the Great Arteries (TGA). Placental transport capabilities appear diminished in HLHS placentas, while microvascular changes are seen in the placentas of fetuses with both TGA and HLHS (Jones et al., 2015; Jones, unpublished data) and appear to be independent of fetal weight, as TGA babies are frequently significantly larger at birth than HLHS babies (Puri et al., 2017; Alsaied et al., 2018).

Previously, we (Jones et al., 2015) noted disruptions in the vascular trees in placentas of babies born with HLHS. Recent histopathological analyses of placentas from babies born with TGA show similar abnormalities, yet the babies generally weighed more at birth than the HLHS cohort (Jones, unpublished data). In addition, Doppler ultrasound abnormalities were absent despite the growth restriction and placental dysregulation (Gembruch et al., 2003; Parra-Saavedra et al., 2013). This suggests that the formation of the vascular tree in the placenta of CHD fetuses is abnormal despite subtype and while the potential for flow disruption and feedback may exist, it may be more likely that this disruption reflects abnormal vascular formation during early pregnancy and leads to the question if this is seen in other vascular beds of the CHD fetus (Sun et al., 2015).

## Future directions

To investigate the involvement of the placenta in congenital heart disease further, the combination of clinical data, human samples, murine models, and *in vitro* multi-cell models will be necessary. Furthermore, moving beyond examining the placenta only at the time of delivery, and utilizing newer technologies for studying the placenta during pregnancy in humans will be vital. Collection of human placental samples as well as the use of samples of maternal blood throughout pregnancy from the multiple subtypes of congenital heart disease will enable us to establish if the common placental perturbations we have already identified are indeed apparent across the spectrum of CHDs, and if those changes can be identified by assessing and characterizing extracellular vesicles within the maternal circulation during pregnancy.

The use of “heart-specific” animal models will enable us to study the progression of placental disruption in association with cardiac malformations at time points in gestation that we cannot access in humans, such as during the early first trimester. Furthermore, using capabilities designed in our lab we will investigate if correcting the identified placental defects during early pregnancy can impact fetal, cardiac and vascular bed development and growth. We hypothesize that very early signaling events impact fetal vasculature, affecting both placenta and heart, and that correcting the signaling pathways via the placenta may offer avenues for potential therapies.

Early signaling mechanisms between the fetus and the placenta remain largely under-investigated but may represent a mechanism that also links heart and placenta development and function. Investigations using co-cultured human cells *in vitro* will again enable us to identify if disturbances in cardiac cells or trophoblast cells can impact each other via the fetal vasculature and endothelium.

At this current time it is impossible to say if, in humans, placental changes may contribute to the cardiac anomalies in congenital heart disease or if the placental changes recently identified are due to a shared disrupted etiology which impact fetal development and growth concurrently. Clearly studies from murine models and chick embryo studies would suggest that placental structural and vascular changes contribute to congenital heart defects as described above. We aim to determine if this is true in humans and if this may provide a path for the potential treatment of CHD *in utero*.

## Ethics statement

This protocol was approved by the Institutional Review Boards of Cincinnati Children's Hospital Medical Center and Good Samaritan Hospital (Cincinnati, Ohio) under study number 2010–2610. Patient data were identified prior to analysis. Animal studies were approved by Cincinnati Children's Hospital Medical Center under IACUC protocol 2015-0078.

## Author contributions

JAC, JC, and HJ contributed to the writing of the manuscript, data generation.

### Conflict of interest statement

The authors declare that the research was conducted in the absence of any commercial or financial relationships that could be construed as a potential conflict of interest. The reviewer AM and handling editor declared their shared affiliation at time of review.

## Footnotes

^1^Centre for Trophoblast Research. *Boyd Collection*. Available online at: https://www.trophoblast.cam.ac.uk/Resources/ndp-index (Accessed May 2, 2018).
