# Supplementary material for: The Role of Abnormal Placentation in Congenital Heart Disease; Cause, Correlate, or Consequence?
Source: Front Physiol. 2018 Aug 7;9:1045. doi: 10.3389/fphys.2018.01045 (PMC6091057; doi:10.3389/fphys.2018.01045)
Supplement: Supplementary file 2 [file Data_Sheet_1.DOCX]

**Supplementary Methods:**

The cohort of patients was assembled retrospectively from a single center case series at Cincinnati Children’s Hospital Medical Center (CCHMC) and Good Samaritan Hospital (GSH, Cincinnati, OH) in 2003-2014. Maternal, fetal, and neonatal clinical data were collected at full term (>37 weeks gestation) from HLHS and TGA cases with no genetic complications, multiple gestation pregnancies, history of maternal diabetes, preeclampsia, or hypertension. TGA was defined as having the aorta arise predominantly from the right ventricle and pulmonary artery from the left ventricle. HLHS were defined as previously published (Jones et al. 2015). Controls were defined as full term births with no maternal or fetal issues. The Institutional Review Boards of CCHMC and GSH approved this study.

**Isolation and analysis of RNA from FFPE samples**

RNA was extracted from formalin-fixed, paraffin-embedded (FFPE) human placenta tissue blocks using the RecoverAll Total Nucleic Acid Isolation kit (Ambion). Three-20um sections were cut from the center of the FFPE blocks and RNA was extracted following manufacturer’s instructions using a 2 hour proteinase K digestion at 50°C. RNA integrity was assessed by DV200 using a Fragment Analyzer (advanced Analytical), specifically to determine the applicability of degraded RNAs to NGS library preparation.

**RNA Sequencing**

RNA sequencing was performed by the University of Cincinnati Genomics, Epigenomics and sequencing core

*Target RNA enrichment*

Ribo-Zero Gold kit (Illumina, San Diego, CA) was used to deplete rRNA before library preparation for RNA-seq. Ribo-Depletion script was run on WaferGen (Fremont, CA) Apollo 324 system toautomate RNA depletion with 1 µg of total RNA as input.

*RNA-seq library preparation*

The RNAseq library was prepared by using NEBNext Ultra Directional RNA Library Prep kit (New England BioLabs, Ipswich, MA). During the second cDNA synthesis dUTP was incorporated into the second cDNA strand to maintain strand specificity. The purified cDNA was end repaired, dA tailed, and ligated to an adapter with a stem-loop structure. The dUTP-labelled 2nd strand cDNA was removed by USER enzyme to maintain strand specificity. The libraries were amplified with PCR and purified alongside the library prep negative controls via AMPure XP beads for QC analysis. The quality and yield of the library were analyzed by Bioanalyzer (Agilent, Santa Clara, CA) using DNA high sensitivity chip. To accurately quantify the library concentration for the clustering, the library was diluted 1:104 in dilution buffer (10 mM Tris-HCl, pH 8.0 with 0.05% Tween 20) and measured by NEBNext Library Quant Kit (New England BioLabs) using QuantStudio 5 Real-Time PCR Systems (Thermo Fisher, Waltham, MA).

*Cluster Generation and HiSeq Sequencing*

To study differential gene expression, individually indexed and compatible libraries were proportionally pooled (~50 million reads per sample in general) for clustering in cBot system (Illumina, San Diego, CA). Libraries at the final concentration of 15 pM were clustered onto a single read (SR) flow cell using Illumina TruSeq SR Cluster kit v3, and sequenced to 50 bp using TruSeq SBS kit on Illumina HiSeq system.

**mRNA-sequence data analyses**

Adapters were trimmed from the reads using CutAdapt V1.8.1(Martin 2011). TopHat V 2.0.13 (Trapnell, Pachter, and Salzberg 2009) and Bowtie V2.1.0(Langmead et al. 2009) were used to align the reads to Human Genome GRCh37 (hg19) and generate BAM files for further analyses. AltAnalyze (Emig et al. 2010) was used to determine differential gene expression between the sample groups, HLHS and term placentas, and HLHS and TGA placentas. Differentially expressed genes were clustered using GO-Elite with a calculated Z-score of 1.96 and p < 0.05 using Fisher Exact Test (Zambon et al. 2012).

**Supplemental Table 1.** To investigate the biological functions, 75 differentially expressed genes (DEGs) from cluster 1 were analyzed in AltAnalyze using GO analysis with a Z-score cutoff of 1.97 and p < 0.05. Important pathways include left/right axis specification and symmetry, heart looping, heart development, tissue remodeling, and ATP synthesis.
